# Trophic interactions and stage-specific accumulation of trace elements in the marine parasitic nematode *Anisakis simplex*

**DOI:** 10.1016/j.crpvbd.2026.100419

**Published:** 2026-07-27

**Authors:** Michelle Musiol, Milen Nachev, Kaan Kumas, Kurt Buchmann, Bernd Sures

**Affiliations:** a Aquatic Ecology and Centre for Water and Environmental Research (ZWU), University of Duisburg-Essen, Universitätsstraße 5, Essen, 45141, Germany; b Research Center One Health Ruhr, Research Alliance Ruhr, University of Duisburg-Essen, Universitätsstraße 5, Essen, 45141, Germany; c Department of Veterinary Disease Biology, Faculty of Health and Medical Sciences, University of Copenhagen, Frederiksberg C, Denmark

**Keywords:** Parasitic nematodes, *Anisakis*, Developmental stages, Metal accumulation, Trophic ecology, Host-parasite interactions, Stable isotopes

## Abstract

Marine ecosystems are increasingly affected by anthropogenic stressors, including trace elements (TE), highlighting the need for sensitive bioindicators. Fish parasites have proven to be effective bioindicators for TE accumulation, while information on TE levels in parasites of marine mammals remains scarce. The present study combined measurements of TE levels in parasites of fish and mammals. We investigated different developmental stages of the same nematode species, *Anisakis simplex*, in their different hosts with trophic interactions of larval and adult stages from their respective hosts, the North Sea herring (*Clupea harengus*) and harbour seals (*Phoca vitulina*), sampled in the North Sea. Both larval and adult *A. simplex* accumulated selected TE at higher levels than their hosts, particularly non-essential elements in seals, resulting in high bioconcentration factors. Adult *A. simplex* exhibited significantly higher concentrations of several essential elements compared to the larval stages, except for Ag, which was accumulated at higher levels in larvae. Stable isotope analysis revealed a consistently lower level of δ^15^N in both larval and adult *A. simplex* relative to host tissues. This pattern suggests that adult *A. simplex* may not primarily feed on host tissues and may instead rely partly on host metabolites, such as ingested food items in the seal stomach. Moreover, elevated δ^13^C indicates the utilization of distinct carbon sources by the parasites compared to their hosts. These results indicate that TE accumulation in *A. simplex* is influenced by both developmental stage and host species, supporting their value as stage-specific bioindicators in marine pollution monitoring.

## Highlights

• Adult *Anisakis simplex* accumulated most trace elements more strongly than larval stages.
• Adult nematodes from seals accumulated mainly non-essential elements at higher levels than their hosts.
• δ^15^N depletion in *A. simplex* suggests that these nematodes do not exclusively feed on the host.
• Trace element accumulation depended on nematode developmental stage and host species.

## Graphical abstract

**Figure:**
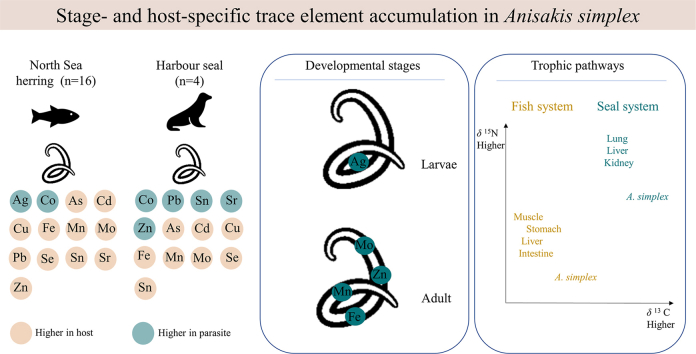

## 1 Introduction

Marine ecosystems are increasingly affected by anthropogenic activities, including the input of trace elements (TE) (Delgado-Suarez et al., 2023; van der Spuy et al., 2023). TE can be divided into essential elements (e.g. iron (Fe), copper (Cu) or zinc (Zn)), which are required for key biochemical processes, and non-essential elements (e.g. cadmium (Cd), lead (Pb) or silver (Ag)), which have no biological function and can be toxic even at low concentrations (Fontaine et al., 2007; Simokon and Trukhin, 2021). Also, essential elements may exert toxic effects when exceeding physiological thresholds (Bryan, 1971), highlighting the need for continuous TE monitoring in marine environments, given their potential for bioaccumulation and biomagnification along food webs. Traditionally, such monitoring has relied on free-living bioindicators, including fishes or marine mammals (dos Santos Lima et al., 2024; Oros, 2025).

In recent years, parasites have increasingly gained attention as complementary bioindicators (Nachev and Sures, 2016; Sures et al., 2017, 2025; Nachev et al., 2025). Numerous studies on fish parasites have demonstrated that helminths, particularly acanthocephalans and cestodes, but also nematodes, can accumulate pollutants to concentrations far exceeding those of their host, rendering them effective bioindicators (Nachev and Sures, 2016; Sures et al., 2017). This capability is linked to their often exceptionally high accumulation capacity, with some species reaching element concentrations thousands of times higher than those of their hosts (Sures et al., 1999; Sures, 2004). In contrast to fish-parasite systems, considerably less is known about TE levels in parasites of marine mammal hosts. A recent study on seals indicated that their parasites can accumulate selected elements at levels that are considerably higher than those observed in their hosts, although these differences appear less pronounced than those in fish-parasite systems, potentially reflecting the enhanced detoxification mechanisms in seals (Musiol et al., 2026a).

Besides the host-specific bioaccumulation patterns in parasites, an important but scarcely studied aspect concerns TE accumulation in the developmental stages of parasites, especially for taxa with complex life cycles (Sures and Taraschewski, 1995; Siddall and Sures, 1998). Stage-specific accumulation patterns are of particular relevance, as larval and adult development stages differ markedly in terms of their host (intermediate, paratenic and definitive hosts) and microhabitat preferences. This may strongly influence physiology (e.g. in homothermic *vs* heterothermic hosts) and feeding mode, and thereby bioavailability and element uptake by parasites within the host (Nachev et al., 2013). In fishes, adult acanthocephalans typically accumulate higher levels of TE than their larval stages (cystacanths, see e.g. Sures and Taraschewski, 1995), which themselves exhibit lower concentrations than their intermediate hosts (Sures et al., 1994; Sures and Taraschewski, 1995; Sures, 2003). Apart from acanthocephalans, studies addressing TE accumulation of both larval and adult parasites remain scarce. One of the few available studies recorded higher levels of Cd, Cu and Zn in adult *Anisakis simplex* compared to larval stages, potentially attributable to the encapsulated and metabolically reduced state of larvae within intermediate fish hosts (Pascual and Abollo, 2003). However, to date, no studies have systematically compared TE accumulation between larval and adult parasitic nematodes obtained from hosts within the same geographical region, leaving a substantial knowledge gap regarding the bioavailability of TE and the mechanisms underlying developmental stage-specific bioaccumulation of nematodes.

In this context, stable isotope analysis (SIA) provides a powerful approach to elucidating trophic relationships and nutritional pathways in host-parasite systems (Thieltges et al., 2019; Nachev et al., 2025). Specifically, knowledge about the feeding modes of nematodes, particularly in marine mammals, remains limited, which hampers the interpretation of TE accumulation patterns. It is, for example, not fully understood whether adult *Anisakis* spp. feed primarily on host tissue or host diet (Mastick et al., 2024). Measurements of stable isotopes of carbon (δ^13^C) and nitrogen (δ^15^N) can be a useful tool for elucidation of feeding mechanisms by providing information on trophic positions and nutrient sources (Thieltges et al., 2019). As adult and larval nematodes inhabit different host tissues and may rely on distinct nutritional sources (Nachev et al., 2013), stable isotope signatures can help to distinguish whether observed differences in TE accumulation reflect dietary uptake, exposure pathways or physiological processes. For example, previous research on *A*. *simplex* L3-larvae showed lower δ^15^N values than their hosts, indicating that larvae do not primarily feed on host tissue but instead reflect isotopic signatures acquired from their previous host (Sabadel et al., 2025). Adult nematodes, in contrast, generally exhibit higher δ^15^N values than their hosts (summarised in Nachev et al., 2025). However, to date, there has been no study comparing stable isotopes in marine adult and larval nematodes to ascertain more about the feeding ecology of seal nematode parasites.

Therefore, the present study aimed to compare TE accumulation and stable isotope signatures in larval and adult stages of *A*. *simplex* and to relate these patterns to their respective hosts. Specifically, the aims of the study were: (i) to investigate the TE accumulation patterns in larval and adult nematodes with respect to tissues of their intermediate and definitive hosts; and (ii) to evaluate differences in the stable isotope composition of nematodes in the context of host transmission and feeding ecology, in order to elucidate the mechanisms underlying observed TE enrichment patterns.

## 2 Materials and methods

### 2.1 Sample collection

Harbour seals (*Phoco vitulina*, *n* = 4) and Atlantic herring (*Clupea harengus*, *n* = 10) collected in Limfjorden in Denmark (Kumas et al., 2025a, 2025b) were frozen and later dissected at the Department of Veterinary and Animal Sciences, University of Copenhagen. The present study examined stage-specific trace element accumulation in seal tissues (kidney, liver, lung, stomach), fish tissues (liver, stomach, intestine, muscle), and in adult and larval stages of *A. simplex*, respectively.

### 2.2 Parasite identification

Adult nematodes collected from the stomachs of seals and 3rd-stage larvae obtained from body cavity organs of Atlantic herring were stored in 96% ethanol. Following removal of the capsule of all L3 larvae, parasites were dissected into three segments: anterior, middle, and posterior. The anterior and posterior segments were used for morphological identification, while the middle segment was reserved for elemental and stable isotope analyses. For the morphological examination, the anterior and posterior sections of each specimen were transferred to a glass beaker with lactic acid for clearing; thereafter, the samples were transferred to a microscope slide and mounted with pre-heated glycerin-gelatine (Buchmann, 2007) and examined under a light microscope at different magnifications. Genus-level identification of the adult parasites was conducted based on morphological features such as the structure of ventriculus, labiae and location of the excretory pore, following Krabbe (1878). Similarly, 3rd-stage larvae were identified according to Val’ter et al. (1982) .

### 2.3 Element analysis

Element analyses were performed following microwave-assisted acid digestion. The samples were digested in Xpress vessels with glass inlays (CEM, Matthews, USA). Up to 287 mg of the host tissues and up to 25 mg of the parasites were weighed into the vessels. Due to the low individual biomass of larval nematodes, pools of up to 8 individual worms were combined and, in some cases, larval nematodes from 2 individual fish were pooled to obtain sufficient sample mass for the trace element analysis. Samples were digested with 4 ml of 65% nitric acid using Mars 6 microwave digestion system (CEM, Matthews, USA) at 180 °C. Following digestion, element concentrations of arsenic (^75^As), cadmium (^111^Cd), cobalt (^59^Co), copper (^65^Cu), iron (^57^Fe), lead (^208^Pb), manganese (^55^Mn), molybdenum (^95^Mo), selenium (^82^Se), silver (^107^Ag), strontium (^88^Sr), tin (^120^Sn) and zinc (^66^Zn) were quantified by inductively coupled plasma mass spectrometry (ICP-MS; PerkinElmer Nexion, 2000; Waltham, MA, USA). Detailed information on instrumental settings, calibration procedures, quality control and sample measurements is provided in Musiol et al. (2026a). To ensure analytical reliability, TE concentrations were validated using the certified elements in DORM5. Certified elements in DORM5 were analysed in ten replicates. Measured concentrations, analytical accuracy and detection limits are presented in Table 1. The analytical accuracy for all elements ranged within ±20%, in accordance with Vogelsang and Hädrich (1998).

**Table 1:** Trace element concentrations in DORM5, including analytical accuracy and detection limits for the ICP-MS analysis.

Element	DORM 5 reference (μg/g)	DORM5 measured (μg/g)	Accuracy [%]	Detection limit (μg/l)
Ag	0.135	0.152 ± 0.014	113	0.0018
As	13.30	13.452 ± 1.504	101	0.2020
Cd	0.148	0.138 ± 0.013	93	0.0015
Co	0.063	0.0536 ± 0.008	85	0.0004
Cu	3.30	3.810 ± 0.540	115	0.1637
Fe	113.0	91.739 ± 9.328	81	6.2534
Mn	1.06	0.946 ± 0.140	89	0.0805
Mo	0.134	0.140 ± 0.019	105	0.0040
Pb	0.058	0.057 ± 0.026	98	0.0353
Se	2.400	2.865 ± 0.135	119	0.0239
Sn	0.077	0.067 ± 0.018	87	0.0135
Sr	9.870	11.023 ± 1.057	112	0.0251
Zn	28.700	34.084 ± 3.088	119	2.0722

### 2.4 Stable isotope analysis

Parasite and host samples (0.3–2.0 mg) were freeze-dried (Heto PowerDry LL3000; Thermo Fisher Scientific, Waltham, USA), homogenized and folded into 4 × 6 mm tin foil capsules for solids (IVA Analysentechnik e.K., Meerbusch, Germany). The nitrogen and carbon composition were measured with an isotope ratio mass spectrometer (IRMS, Isoprime visION, Elementar, Germany) connected to an elemental analyzer (EA, Vario ISOTOPE Select, Elementar, Germany). Isotope ratios were expressed in δ notation as the deviation of the isotope ratio of the sample from that of international reference standards. Detailed methodological and instrumental information is provided in Nachev et al. (2017). The samples were measured in triplicate; however, some parasite samples were measured as duplicates or as a single sample, due to limited sample material. Acetanilide (AcAn, pro analysis, Merck, Germany) was used as an internal laboratory standard and was normalized against two international standards, USGS40 and USGS41a (both International Atomic Energy Agency, Vienna).

### 2.5 Data analysis

Data visualization and statistical analyses were conducted using R Software (v.4.3.2; R Core Team, 2023) within RStudio (v.2023.12.0 + 369, RStudio Team, 2022). Graphical outputs and data processing were generated using the packages *ggplot* v.2 4.0.1 (Wickham, 2016), *readxl* v.1.4.3 (Wickham and Bryan, 2023), *dplyr* v.1.1.4 (Wickham et al., 2023), *tidyverse* v.2.0.0 (Wickham et al., 2019), *tidyr* v.1.3.1 (Wickham et al., 2026), *rstatix* v.0.7.2 (Kassambara, 2023). Differences in TE accumulation and stable isotope values between hosts and parasites, as well as among the development stages, were assessed using the Kruskal-Wallis test. Only groups with a minimum of two observations were included in the analysis. For cases where the Kruskal-Wallis test indicated a significant difference, *post-hoc* pairwise comparisons were conducted using Dunn’s tests with false discovery rate (FDR) correction according to Benjamini-Hochberg. FDR correction was applied separately for each element across all pairwise comparisons. Adjusted *P*-values < 0.05 were considered statistically significant. Significance levels were reported as: ***P < 0.001, **P < 0.01, *P < 0.05, and ns for *P* ≥ 0.05.

Bioconcentration factors (BCF) were calculated according to Sures et al. (1999) as follows: BCF = Concentration in parasite/Concentration in host tissue.

## 3 Results

### 3.1 Morphological and molecular identification of the parasites

Based on morphological structures, both adult and larval nematodes were assigned to the genus *Anisakis*. Although molecular identification was not performed in the present study, previous studies conducted molecular identification of both adult (Kumas et al., 2025a) and larval (Kumas et al., 2025b) nematodes collected from the same batches of host species and capture area. These studies identified *A*. *simplex* (*sensu stricto*) based on mitochondrial cytochrome *c* oxidase subunit 2 (*cox*2) and nuclear internal transcribed spacer (ITS) regions. The GenBank accession numbers for the adult nematodes identified as *A. simplex* (*s.s*.) are PQ860936-PQ860960 (ITS) and PV199995-PV200021 (*cox*2). For larval *A. simplex* (*s.s*.), the GenBank accession numbers are PV696447-PV696456 (ITS) and PV711120-PV711129 (*cox*2).

### 3.2 Trace element accumulation in *Anisakis simplex* and host tissues across host species and parasite developmental stages

All four harbour seals and all ten North Sea herrings were infected with nematodes. Specimens of *A. simplex* were recovered from the body cavity of fish and from the stomach of seals. No other parasite species were considered or included in the analysis.

**Fig. 1:**
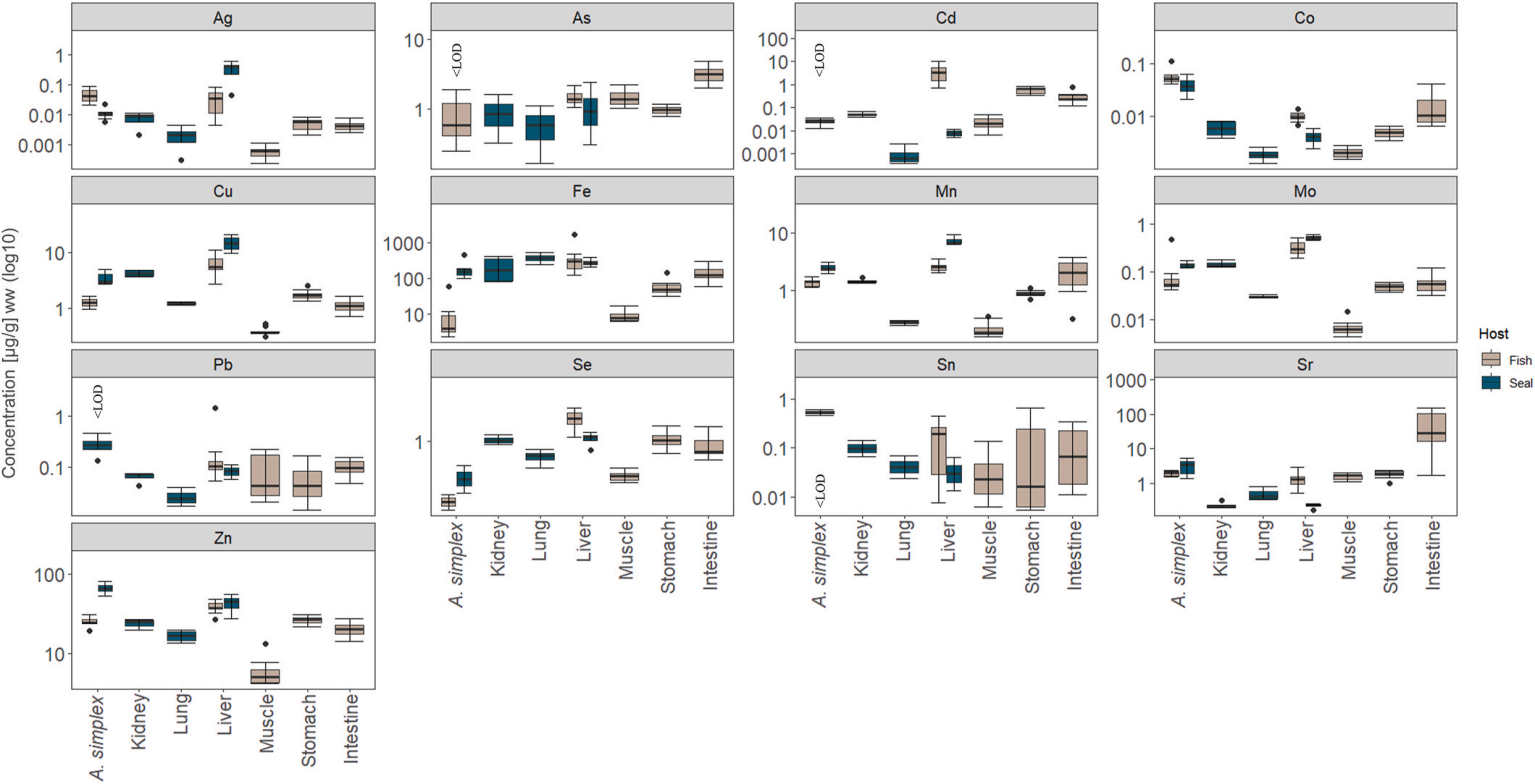
Trace element accumulation in tissues of seals (*n* = 4) and fish (*n* = 10) and their parasite *Anisakis simplex*. Values below the detection limit are indicated as < LOD.

In seals, concentrations of Cd, Co, Mn, Pb, Sr and Zn were significantly higher in adult *A. simplex* than in most host tissues (Fig. 1, Supplementary Table S1). In contrast, in fish hosts, Ag, Co, Mn, Mo and Zn levels were found in significantly higher concentrations in larval *A. simplex* compared to most host tissues (Fig. 1, Supplementary Table S2).

TE concentrations further differed between the two host species. Specifically, Ag, Cu, Mn and Mo concentrations were significantly higher in seal tissues in comparison to fish tissues, whereas the latter accumulated Cd, Co, Se and Sr to a higher extent (Fig. 1, Supplementary Table S3).

**Fig. 2:**
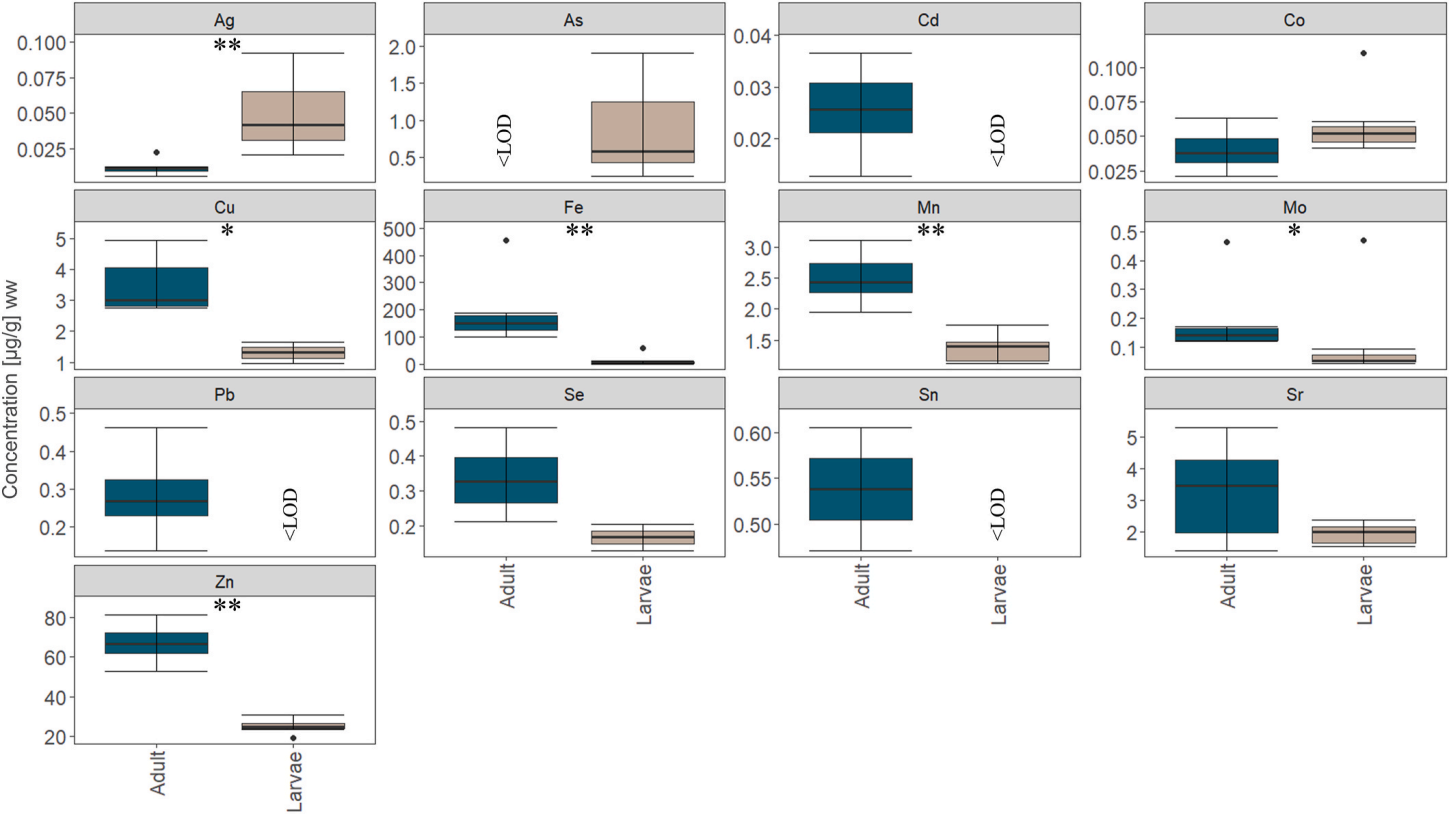
Trace element accumulation in adult and larval *Anisakis simplex*. Significance levels are indicated as follows: *P < 0.05; **P < 0.01; ***P < 0.001. Values below the detection limit are indicated as < LOD.

In addition to host-specific differences, TE enrichment also varied between parasite developmental stages. Adult nematodes exhibited significantly higher concentrations of Fe, Mn, Mo and Zn than larvae, whereas the larvae accumulated Ag at higher levels (Fig. 2, Supplementary Table S4).

### 3.3 Bioconcentration factors

**Fig. 3:**
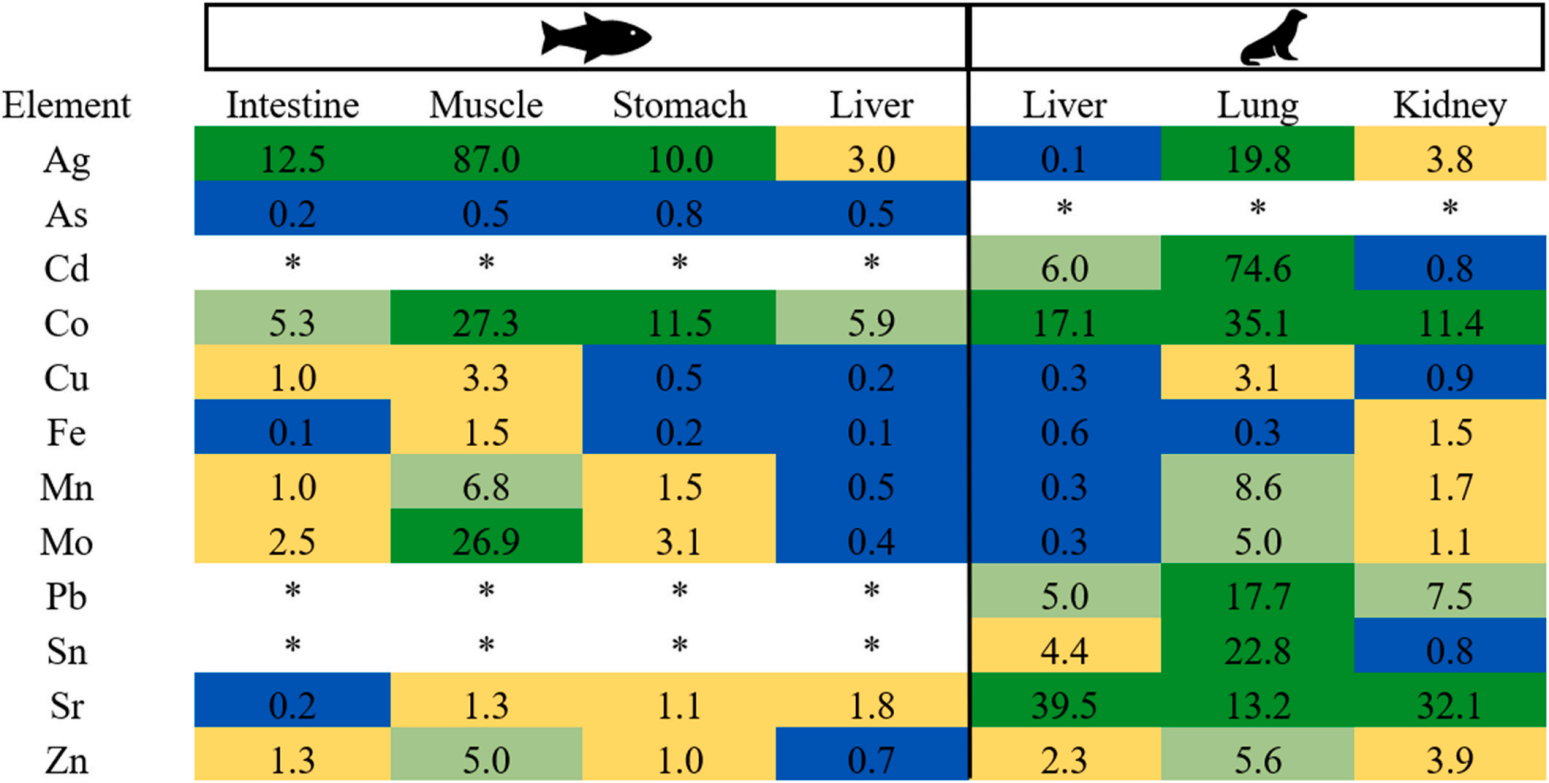
Bioconcentration factors (BCFs) for fish and seals. Values marked with asterisks indicate that the TE concentrations in the parasites were below the detection limit. Colors represent BCF ranges: blue (< 1), orange (1–5), light green (> 5–10), and dark green (> 10).

The bioconcentration factors (BCFs) further highlighted the differences in accumulation patterns between parasites from fish and seals. In fish, the BCFs for As were consistently below one compared to all host tissues, while Ag and Co showed BCFs above one across all host tissues, indicating pronounced accumulation in parasites. The highest BCF in fish (87.0) was observed for Ag in comparison to muscle tissue (Fig. 3). In seals, the BCFs for Co, Pb, Sr and Zn exceeded one compared to all host tissues, indicating accumulation of particularly non-essential elements in the parasites. The highest value (74.6) was recorded for Cd in comparison to the lung tissue (Fig. 3). For both host species, BCFs were generally lowest when compared to liver tissues and highest compared to muscle in fish and lung in seals (Fig. 3).

As the liver was the only tissue analysed in both host species, liver-related BCFs were examined separately to allow direct interspecific comparison. In fish, parasites generally exhibited low to moderate accumulation relative to the liver tissue, with most BCFs below 2, only Ag and Co showed elevated BCFs. Non-essential elements such Cd, Pb and Sn were below the detection limits in parasites, and therefore BCFs for those elements could not be calculated (Fig. 3).

In contrast, parasites from seals exhibited BCFs for non-essential elements with respect to the liver, with elements such as Cd, Pb, Sn and Sr showing values up to 39. The BCFs of seals calculated for the essential elements showed, in general, lower values; however, they remained consistently higher than those observed in fish parasites (Fig. 3).

### 3.4 Stable isotope patterns in selected host-parasite systems

**Fig. 4:**
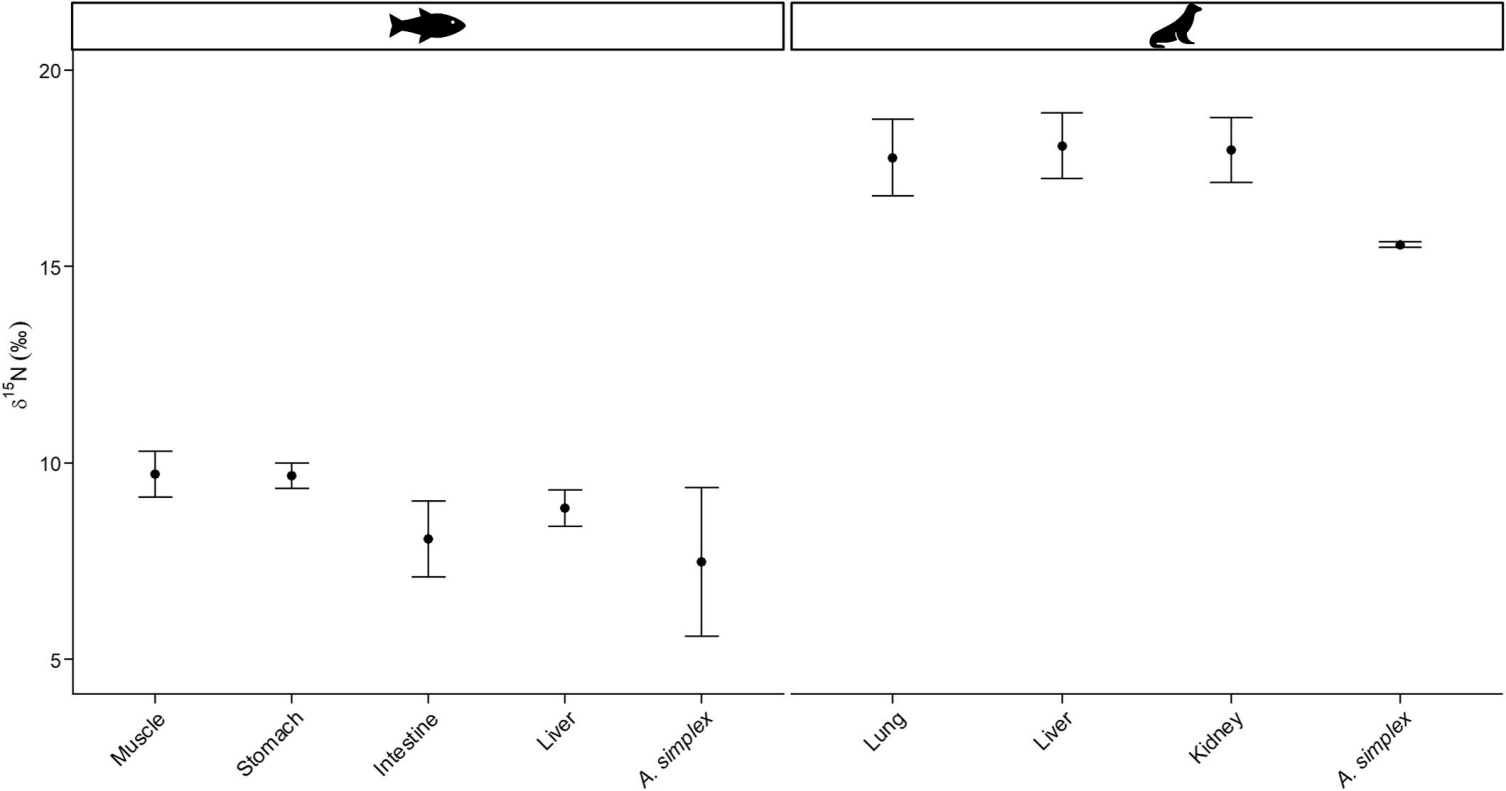
Means and standard deviations of δ^15^N values for fish and seal tissues as well as their parasite *Anisakis simplex*.

For nitrogen, nematodes were depleted in δ^15^N values compared to their hosts. This depletion was statistically significant in fish hosts, whereas in seals the same trend was observed without being statistically significant (Fig. 4, Supplementary Tables S5 and S6).

Clear differences were detected between the two host species. Overall, seals and their parasites showed significantly higher *δ*^15^N ratios compared to fish and their parasites, reflecting the shift in trophic levels between herring and seals (Fig. 4, Supplementary Table S7). This is further supported by the substantially higher δ^15^N values in seal liver compared with fish liver (Difference = 9.21‰, Fig. 4, Supplementary Tables S7 and S8).

Interestingly, this pattern was also shown for the isotopic shift between larval nematodes in fish and adult nematodes in seals. Adult *A. simplex* showed enrichment with heavier nitrogen compared to the larvae (Difference = 8.08‰, Fig. 4, Supplementary Tables S7 and S8).

**Fig. 5:**
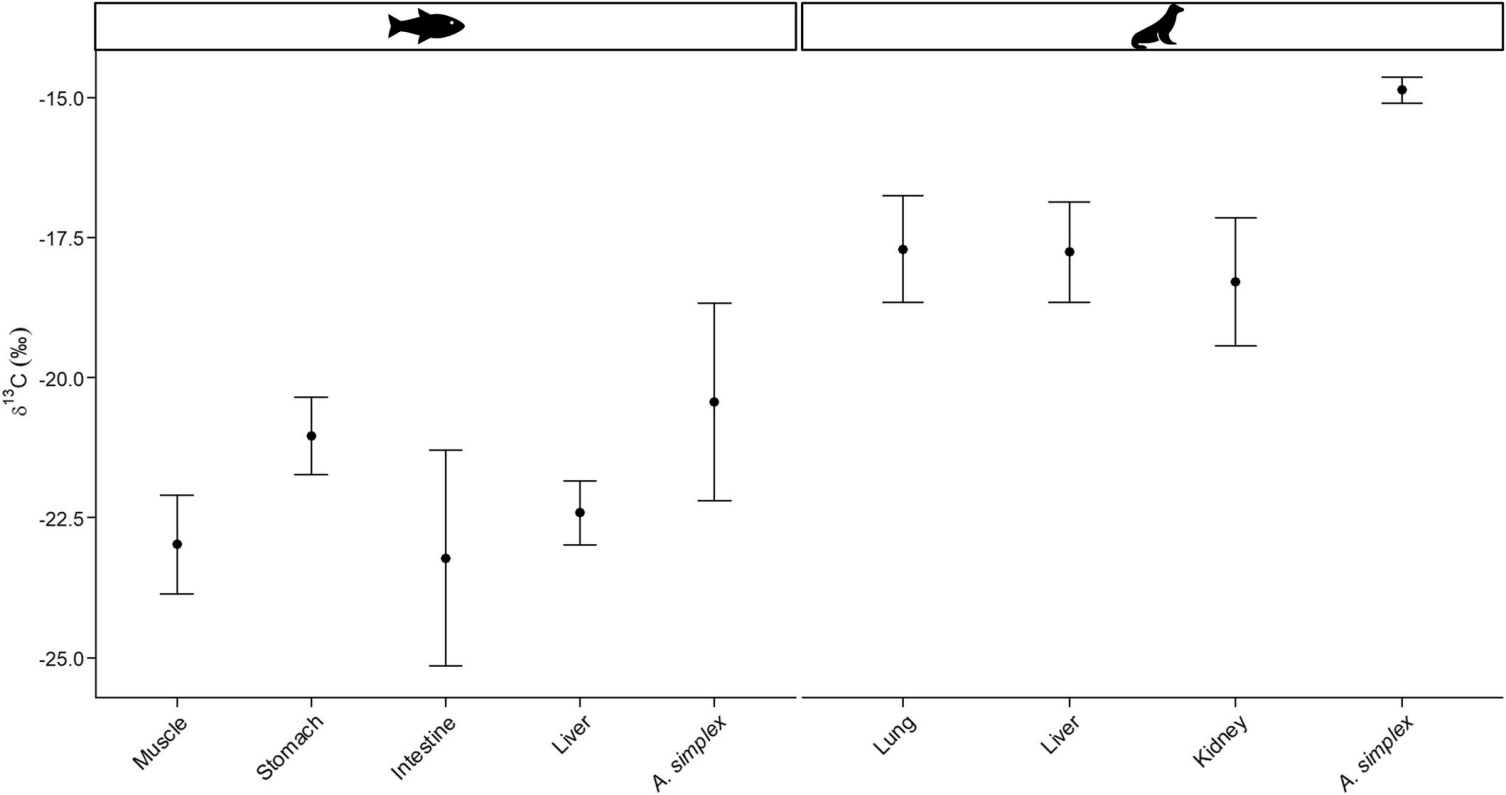
Means and standard deviations of δ^13^C values for fish and seal tissues as well as their parasite *Anisakis simplex*.

A similar host-related pattern was observed for carbon isotopes, although with an opposite direction of fractionation between host and parasite. In both hosts, nematodes were significantly enriched in δ^13^C related to the host tissues, with Δδ^13^C up to 2.79‰ for fish and 3.42‰ in seals, indicating that nematodes of the seals were particularly enriched in heavier carbon (Fig. 5, Supplementary Tables S5, S6 and S8). As observed for nitrogen, adult nematodes exhibited higher δ^13^C values than the larval (Difference = 5.57‰, Fig. 5, Supplementary Tables S7 and S8), again highlighting the isotopic shift associated with host transition.

Similar to nitrogen, seal tissues and their parasites were enriched in carbon compared to fish and their parasites, again reflecting the different trophic positions (Fig. 5).

## 4 Discussion

This study provides new insights into TE enrichment in larval and adult stages of *A. simplex*, highlighting accumulation differences between hosts and parasites, as well as among developmental stages of the nematodes. Furthermore, by addressing the isotope composition of carbon and nitrogen in nematodes in the context of host transmission and their feeding ecology, we aimed to elucidate the mechanisms underlying the TE enrichment patterns.

The comparison between host species and their parasites revealed pronounced accumulation differences between larval nematodes in fish and adult nematodes infecting seals. Generally, larval nematodes exhibited lower TE concentrations than adults. This was also reflected in BCFs, which were consistently higher in seal parasites, particularly for non-essential elements such as Cd, Pb and Sr. The direct comparison between adult and larval stomach nematodes further supports this pattern. Only Ag was elevated in the larvae, whereas Cu, Fe, Mn, Mo and Zn were significantly higher in the adult nematodes.

These differences might be attributed to the different microhabitats and lifestyles of the respective developmental stages. Adult *A. simplex* inhabit the gastrointestinal tract of their definitive hosts, where they are metabolically active, whereas the larvae are typically surrounded by a larval sheath and further encapsulated within host tissues and largely immobile with reduced metabolic activity (Aibinu et al., 2019; Sugaya et al., 2019; Kumas et al., 2024). Under such conditions, TE uptake by larvae is likely dominated by passive absorption across the cuticle, which is less complex than in adults (Nachev et al., 2013). In addition, TE concentrations in larvae may partly reflect exposure during earlier life stages prior to encapsulation.

The elevated TE concentrations observed in *A. simplex* compared to their hosts support previous findings that marine parasites can serve as bioindicators for TE enrichment (Nachev and Sures, 2016; Musiol et al., 2026a). This pattern was further reflected in the high BCFs observed for several elements, indicating that parasites accumulate certain TE at higher levels, exceeding those of their hosts.

In *A. simplex* from seals, elevated concentrations of Cd, Co, Mn, Pb, Sr and Zn were observed. These findings partially contrast with previous results reporting elevated concentrations primarily for As and Co in nematodes from harbour seals (Musiol et al., 2026a). These discrepancies may be related to differences in parasite species composition among the studies. For instance, Musiol et al. (2026a) included other species of nematodes, i.e. *Contracaecum osculatum*, *Hysterothylacium aduncum* and *Pseudoterranova decipiens*, which differ in life cycles and host associations (Lakemeyer et al., 2020). In particular, *H. aduncum* uses fish as both intermediate and definitive (Køie, 1993) and occurs only as L3-larvae in marine mammals, suggesting that these hosts may represent dead-end hosts (Lakemeyer et al., 2020; Pawlak, 2021). Differences in life cycle characteristics and host association may therefore influence TE accumulation patterns. Additionally, host-related factors such as age, sex and nutritional status can further influence the TE accumulation (Watanabe et al., 1998; Agusa et al., 2011; MacMillan et al., 2022; Musiol et al., 2026a).

The accumulation of non-essential elements such as Cd and Pb in seal parasites further indicates that nematodes can serve as bioindicators for both essential and non-essential elements. Previous studies have suggested that nematodes predominantly accumulate essential elements due to their feeding on host tissues (Sures et al., 2017). However, most of these studies focused on nematodes infecting fish hosts (summarised in Sures et al., 2025), whereas research on TE enrichment in parasites of marine mammals is limited. Recent findings from nematodes of harbour porpoises also indicate elevated concentrations of both essential and non-essential elements in nematodes compared to their hosts (Musiol et al., 2026b), supporting the results of the present study.

In fish hosts, larval nematodes exhibited elevated Ag and Co concentrations compared to their host tissues. Correspondingly, BCFs for Ag were particularly high, with values of parasite exceeding those of muscle tissue by up to 87 times, whereas liver-related BCFs were generally lower. Although comparable studies on North Sea herring are lacking, a previous study on *A. simplex* from fish hosts (*Micromesistius poutassou* and *Trachurus trachurus*) from Galicia reported higher concentrations of Cd, Cu, Pb and Zn in nematodes than in their hosts (Pascual and Abollo, 2003). Similar accumulation patterns have also been observed for other nematode species, including *Contracaecum* spp., supporting the general suitability of nematodes as bioindicators for both essential and non-essential elements (Sures, 2003; Thabit, 2023). Differences among studies may reflect variations in host species and geographical region (Kress et al., 1999; Xia et al., 2019).

Distinct TE concentration differences were also detected between the host species, underscoring the influence of host physiology, trophic position, metabolic regulation and habitat on element accumulation. Seals accumulated higher concentrations of Ag, Cu, Mn and Mo in the liver compared to fish, which may reflect bioaccumulation associated with higher trophic level and longer life span of marine mammals (Hansen et al., 2016; Rechimont et al., 2025). Consistent with this interpretation, the δ^15^N values were significantly higher in seals compared to fish, further confirming their position as top predators in the marine food web. In addition to trophic position, element accumulation may also be influenced by feeding habitat. For instance, elevated Ag concentrations have been associated with benthic organisms, suggesting the consumption of benthic prey may contribute to enhanced Ag enrichment in seals (Dehn, 2005). Conversely, fish exhibited higher Cd, Co, Se and Sr concentrations, which may primarily reflect dietary uptake. For instance, Cd and Se are predominantly absorbed through food intake (Xu and Wang, 2002), while Sr is known to accumulate in fish tissues such as liver and gills (Đikanović et al., 2016). The depletion of δ^13^C in fish showed different carbon sources between the host species, indicating different feeding, which would explain the differences in TE enrichment.

Stable isotope analysis also revealed trophic shifts between the host species and parasites, providing insights into the parasite feeding ecology. In fish, larval nematodes are encapsulated and metabolically less active (Kumas et al., 2024), whereas in seals, adult nematodes are metabolically active and likely ingest host-derived material and food items ingested by the seal. Due to their higher energy demands, adult nematodes may ingest higher amounts of nutrients and, consequently, TE. Although the precise feeding mechanisms of adult *A. simplex* within their definitive hosts remain incompletely understood (Mastick et al., 2024), it has been hypothesised that nematodes with buccal capsules, such as *P. decipiens*, feed on mucosal fluids, cell debris and products of the host’s digestion (Zintl et al., 2024). The results of the SIA in the present study are consistent with this hypothesis, indicating that adult *A. simplex* likely derive nutrients primarily from the host’s digestive contents rather than directly feed on the host tissues.

The δ^13^C analysis showed significantly higher values in both adult and larval nematodes compared to the host tissue. These results contrast with previous findings showing lower δ^13^C values in adult nematodes compared to their hosts (Zintl et al., 2024). However, a comparative analysis across more than 100 host-parasite comparisons demonstrated that the Δ^13^C can vary from −8.2 to 6.5‰, indicating that parasites can be enriched in carbon compared to their hosts (Thieltges et al., 2019). The elevated δ^13^C values may reflect uptake of specific dietary components or absorption during earlier life stages.

A limitation of the present study is the relatively small sample size, particularly for harbour seals, which reduces the statistical power of the analysis. Although consistent trends in accumulation were observed, caution is advised when generalising these results to large populations. Future studies with a large sample size across multiple hosts and geographical regions would help to validate and expand upon the observed patterns.

## 5 Conclusions

This study demonstrates that *A. simplex* can accumulate both essential and non-essential elements at concentrations exceeding those of their fish and seal hosts, underscoring their function as effective bioindicators for TE. The accumulation patterns differed markedly between larval and adult stages, indicating that TE uptake is strongly influenced by the parasite life cycle and feeding strategy. Stable isotope analysis further suggests that adult *A. simplex* primarily obtain nutrients from host-derived stomach contents, providing insights into the trophic relationships between parasites and hosts, as well as potential pathways of TE incorporation. These findings highlight the importance of considering parasite developmental stages and host species when assessing TE dynamics in marine host-parasite systems. Further studies should focus on the precise mechanisms of diet intake and TE uptake and retention across developmental stages of parasites. Such research will provide a deeper understanding of parasite life cycles and mechanisms underlying TE accumulation.

## Ethical approval

Not applicable. The present study did not include any handling or experimentation with live animals. The fish and seals examined were received as dead animals landed by local fishermen and treated respectfully and with extreme care to prevent any damage of tissues.

## Statement on the use of AI-assisted technologies

During the preparation of this paper, the authors occasionally used ChatGPT to improve the readability and language of the manuscript, paraphrase, and check grammar and spelling. All content generated by the tool was reviewed, revised, and verified by the authors, who take full responsibility for the integrity and accuracy of the work and the content of this published article.

## CRediT authorship contribution statement

**Michelle Musiol:** Methodology, Formal analysis, Visualization, Writing - original draft, Writing - review & editing. **Milen Nachev:** Methodology, Validation, Writing - review & editing. **Kaan Kumas:** Investigation, Methodology, Writing - review & editing. **Kurt Buchmann:** Investigation, Methodology, Conceptualization, Resources, Writing - review & editing. **Bernd Sures:** Conceptualization, Resources, Supervision, Funding acquisition, Writing - review & editing.

## Funding

The analysed samples belonging to the study SEAL received support from the 10.13039/100008396Danish Ministry of Food, Agriculture and Fisheries and the European Fund (EHFAF EFVMB-23-0034).

## Declaration of competing interests

The authors declare that they have no known competing financial interests or personal relationships that could have appeared to influence the work reported in this paper.

## Data Availability

The data supporting the conclusions of this article are included within the article and its supplementary file.

## References

[bib1] Agusa T., Yasugi S., Iida A., Ikemoto T., Anan Y., Kuiken T. (2011). Accumulation features of trace elements in mass-stranded harbor seals (*Phoca vitulina*) in the North Sea coast in 2002: the body distribution and association with growth and nutrition status. Mar. Pollut. Bull..

[bib2] Aibinu I.E., Smooker P.M., Lopata A.L. (2019). Anisakis nematodes in fish and shellfish - from infection to allergies. Int. J. Parasitol.: Parasites Wildl..

[bib3] Bryan G.W. (1971). The effects of heavy metals (other than mercury) on marine and estuarine organisms. Proc. R. Soc. Lond. B Biol. Sci..

[bib4] Buchmann K. (2007).

[bib5] Dehn L.A. (2005).

[bib6] Delgado-Suarez I., Lozano-Bilbao E., Hardisson A., Paz S., Gutiérrez Á.J. (2023). Metal and trace element concentrations in cetaceans worldwide: a review. Mar. Pollut. Bull..

[bib8] dos Santos Lima G., Pedrobom J.H., Suarez C.A., Torres-Florez J.P., Vidal L.G., Domit C., Menegario A.A. (2024). Bioaccumulation of trace elements in marine mammals: new data and transplacental transfer on threatened species. Sci. Total Environ..

[bib9] Fontaine M.C., Tolley K.A., Siebert U., Gobert S., Lepoint G., Bouquegneau J.M., Das K. (2007). Long-term feeding ecology and habitat use in harbour porpoises *Phocoena phocoena* from Scandinavian waters inferred from trace elements and stable isotopes. BMC Ecol..

[bib10] Hansen A.M.K., Bryan C.E., West K., Jensen B.A. (2016). Trace element concentrations in liver of 16 species of cetaceans stranded on Pacific Islands from 1997 through 2013. Arch. Environ. Contam. Toxicol..

[bib7] Đikanović V., Skorić S., Gačić Z. (2016). Concentrations of metals and trace elements in different tissues of nine fish species from the Međuvršje Reservoir (West Morava River Basin, Serbia). Arch. Biol. Sci..

[bib11] Kassambara A. (2023). rstatix: pipe-friendly framework for basic statistical tests. R package version 0.7.2.

[bib12] Køie M. (1993). Aspects of the life cycle and morphology of *Hysterothylacium aduncum* (Rudolphi, 1802) (Nematoda, Ascaridoidea, Anisakidae). Can. J. Zool..

[bib13] Krabbe H. (1878). Sælernes og tandhvalernes spolorme. Kgl. Danske. Vidensk. Selsk. Forh.

[bib14] Kress N., Herut B., Shefer E., Hornung H. (1999). Trace element levels in fish from clean and polluted coastal marine sites in the Mediterranean Sea, Red Sea and North Sea. Helgol. Mar. Res..

[bib15] Kumas K., Al-Jubury A., Kania P.W., Abusharkh T., Buchmann K. (2024). Location and elimination of *Anisakis simplex* third-stage larvae in Atlantic herring *Clupea harengus* L. Int. J. Parasitol.: Parasites Wildl..

[bib16] Kumas K., Andersen M.H., Jaafar R., Henard C., Kania P.W., Buchmann K. (2025). Protective immune response in rainbow trout (*Oncorhynchus mykiss*) against the parasitic nematode *Anisakis simplex*. Front. Immunol..

[bib17] Kumas K., Gonzalez C.M.F., Kania P.W., Buchmann K. (2025). Gastrointestinal parasites of harbour seal (*Phoca vitulina* L.) in Danish marine waters: prevalence, abundance, intensity and reproductive potential. Int. J. Parasitol. Parasites Wildl..

[bib18] Lakemeyer J., Siebert U., Abdulmawjood A., Ryeng K.A., Ijsseldijk L.L., Lehnert K. (2020). Anisakid nematode species identification in harbour porpoises (*Phocoena phocoena*) from the North Sea, Baltic Sea and North Atlantic using RFLP analysis. Int. J. Parasitol. Parasites Wildl..

[bib19] MacMillan G.A., Amyot M., Daoust P.Y., Lemire M. (2022). Age-specific trace element bioaccumulation in grey seals from the Gulf of St. Lawrence. Chemosphere.

[bib20] Mastick N.C., Fiorenza E., Wood C.L. (2024). Meta-analysis suggests that, for marine mammals, the risk of parasitism by anisakids changed between 1978 and 2015. Ecosphere.

[bib21] Musiol M., Nachev M., Striewe L.C., Lehnert K., Siebert U., Sures B. (2026). Trace element accumulation in parasites of grey seals and harbour seals from the North Sea and Baltic Sea. Mar. Pollut. Bull..

[bib22] Musiol M., Nachev M., Striewe L.C., Lehnert K., Siebert U., Sures B. (2026). Accumulation of trace elements in harbour porpoises and their selected helminths: a study from the North Sea and the Baltic Sea. Environ. Pollut..

[bib23] Nachev M., Jochmann M.A., Walter F., Wolbert J.B., Schulte S.M., Schmidt T.C., Sures B. (2017). Understanding trophic interactions in host-parasite associations using stable isotopes of carbon and nitrogen. Parasites Vectors.

[bib24] Nachev M., Riekenberg P.M., Jochmann M.A., Born-Torrijos A., van der Meer M.T., Smit, Smit N.J., Sures B. (2025). Aquatic Parasitology: Ecological and Environmental Concepts and Implications of Marine and Freshwater Parasites.

[bib25] Nachev M., Schertzinger G., Sures B. (2013). Comparison of the metal accumulation capacity between the acanthocephalan *Pomphorhynchus laevis* and larval nematodes of the genus *Eustrongylides* sp. infecting barbel (*Barbus barbus*). Parasites Vectors.

[bib26] Nachev M., Sures B. (2016). Environmental parasitology: parasites as accumulation bioindicators in the marine environment. J. Sea Res..

[bib27] Oros A. (2025). Bioaccumulation and trophic transfer of heavy metals in marine fish: ecological and ecosystem-level impacts. J. Xenobiot..

[bib28] Pascual S., Abollo E. (2003). Accumulation of heavy metals in the whaleworm *Anisakis simplex s.l.* (Nematoda: Anisakidae). J. Mar. Biol. Assoc. U. K..

[bib29] Pawlak J. (2021). *In situ* evidence of the role of *Crangon crangon* in infection of cod *Gadus morhua* with nematode parasite *Hysterothylacium aduncum* in the Baltic Sea. Parasitology.

[bib30] R Core Team (2023). https://www.R-project.org/.

[bib31] Rechimont M.E., Amezcua F., Ruelas-Inzunza J.R., Cruz-Garcìa R., Vallarta-Zárate J.R.F., Amezcua-Linares F. (2025). Mercury and selenium trophic transfer in the Mexican California current ecosystem using a top predator as a model. Fishes.

[bib32] RStudio Team (2022).

[bib33] Sabadel A., Gay M., Lane H.S., Bourgau O., Bury S.J., Delgado J., Duflot M. (2025). Just hitching a ride: stable isotopes reveal non-feeding behaviour of *Anisakis simplex* within its host fish. J. Fish. Dis..

[bib34] Siddall R., Sures B. (1998). Uptake of lead by *Pomphorhynchus laevis* cystacanths in *Gammarus pulex* and immature worms in chub (*Leuciscus cephalus*). Parasitol. Res..

[bib35] Simokon M.V., Trukhin A.M. (2021). Analysis of essential and non-essential trace elements in the organs of a mother-fetus pair of spotted seals (*Phoca largha*) from the Sea of Japan. Environ. Sci. Pollut. Res. Int..

[bib36] Sugaya S., Takizawa Y., Chikaraishi Y. (2019). What is the parasitic nematode *Anisakis* doing in host fish?. Res. Org. Geochem..

[bib37] Sures B. (2003). Accumulation of heavy metals by intestinal helminths in fish: an overview and perspective. Parasitology.

[bib38] Sures B. (2004). Environmental parasitology: relevancy of parasites in monitoring environmental pollution. Trends Parasitol..

[bib39] Sures B., Nachev M., Grabner D., Wepener V., Zimmermann S., Smit N.J., Sures B. (2025). Aquatic Parasitology: Ecological and Environmental Concepts and Implications of Marine and Freshwater Parasites.

[bib40] Sures B., Nachev M., Selbach C., Marcogliese D.J. (2017). Parasite responses to pollution: what we know and where we go in environmental parasitology. Parasites Vectors.

[bib41] Sures B., Siddall R., Taraschewski H. (1999). Parasites as accumulation indicators of heavy metal pollution. Parasitol. Today.

[bib43] Sures B., Taraschewski H., Jackwerth E. (1994). Comparative study of lead accumulation in different organs of perch (*Perca fluviatilis*) and its intestinal parasite *Acanthocephalus lucii*. Bull. Environ. Contam. Toxicol..

[bib42] Sures B., Taraschewski H. (1995). Cadmium concentrations in two adult acanthocephalans, *Pomphorhynchus laevis* and *Acanthocephalus lucii*, as compared with their fish hosts and cadmium and lead levels in larvae of *A. lucii* as compared with their crustacean host. Parasitol. Res..

[bib44] Thabit H. (2023). Biomonitoring of heavy metals using *Contracaecum quadripapillatum* (Nematoda) in comparison to its fish host, *Lates niloticus*, from the Nile River, Egypt. Environ. Monit. Assess..

[bib45] Thieltges D.W., Goedknegt M.A., O’Dwyer K., Senior A.M., Kamiya T. (2019). Parasites and stable isotopes: a comparative analysis of isotopic discrimination in parasitic trophic interactions. Oikos.

[bib46] Val’ter E.D., Popova T.I., Valovaya M.A. (1982). Scanning electron microscope study of four species of anisakid larvae (Nematoda: Anisakidae). Helminthologia.

[bib47] van der Spuy L., Erasmus J.H., Nachev M., Schaeffner B.C., Sures B., Wepener V., Smit N.J. (2023). The use of fish parasitic isopods as element accumulation indicators in marine pollution monitoring. Mar. Pollut. Bull..

[bib48] Vogelsang J., Hädrich J. (1998). Limits of detection, identification and determination: a statistical approach for practitioners. Accred Qual. Assur..

[bib49] Watanabe I., Tanabe S., Amano M., Miyazaki N., Petrov E.A., Tatsukawa R. (1998). Age-dependent accumulation of heavy metals in Baikal seal (*Phoca sibirica*) from the Lake Baikal. Arch. Environ. Contam. Toxicol..

[bib50] Wickham H. (2016).

[bib51] Wickham H., Averick M., Bryan J., Chang W., McGowan L.D., François R. (2019). Welcome to the tidyverse. J. Open Source Softw..

[bib53] Wickham H., François R., Henry L., Müller K., Vaughan D. (2023). dplyr: a grammar of data manipulation. R package version 1.1.4.

[bib54] Wickham H., Vaughan D., Girlich M. (2026). tidyr: tidy messy data. https://tidyr.tidyverse.org.

[bib52] Wickham H., Bryan J. (2023). readxl: read Excel files. R package version 1.4.3.

[bib55] Xia W., Chen L., Deng X., Liang G., Giesy J.P., Rao Q. (2019). Spatial and interspecies differences in concentrations of eight trace elements in wild freshwater fishes at different trophic levels from middle and eastern China. Sci. Total Environ..

[bib56] Xu Y., Wang W.X. (2002). Exposure and potential food chain transfer factor of Cd, Se and Zn in marine fish *Lutjanus argentimaculatus*. Mar. Ecol. Prog. Ser..

[bib57] Zintl A., Imlau M., Schertzer J., Zhang H., Saint-Marc A., Schmidt O. (2024). Use of stable isotope ratio analysis to investigate the biology and clinical significance of seal parasites. Parasitology.

